# The Social Construction of Categorical Data: Mixed Methods Approach to Assessing Data Features in Publicly Available Datasets

**DOI:** 10.2196/59452

**Published:** 2025-01-28

**Authors:** Theresa Willem, Alessandro Wollek, Theodor Cheslerean-Boghiu, Martha Kenney, Alena Buyx

**Affiliations:** 1 Institute of History and Ethics in Medicine School of Medicine and Health Technical University of Munich Munich Germany; 2 Helmholtz AI Helmholtz Munich Munich Germany; 3 Munich Institute of Biomedical Engineering School of Computation, Information, and Technology Technical University of Munich Munich Germany; 4 Women & Gender Studies San Francisco State University San Francisco, CA United States

**Keywords:** machine learning, categorical data, social context dependency, mixed methods, dermatology, dataset analysis

## Abstract

**Background:**

In data-sparse areas such as health care, computer scientists aim to leverage as much available information as possible to increase the accuracy of their machine learning models’ outputs. As a standard, categorical data, such as patients’ gender, socioeconomic status, or skin color, are used to train models in fusion with other data types, such as medical images and text-based medical information. However, the effects of including categorical data features for model training in such data-scarce areas are underexamined, particularly regarding models intended to serve individuals equitably in a diverse population.

**Objective:**

This study aimed to explore categorical data’s effects on machine learning model outputs, rooted the effects in the data collection and dataset publication processes, and proposed a mixed methods approach to examining datasets’ data categories before using them for machine learning training.

**Methods:**

Against the theoretical background of the social construction of categories, we suggest a mixed methods approach to assess categorical data’s utility for machine learning model training. As an example, we applied our approach to a Brazilian dermatological dataset (Dermatological and Surgical Assistance Program at the Federal University of Espírito Santo [PAD-UFES] 20). We first present an exploratory, quantitative study that assesses the effects when including or excluding each of the unique categorical data features of the PAD-UFES 20 dataset for training a transformer-based model using a data fusion algorithm. We then pair our quantitative analysis with a qualitative examination of the data categories based on interviews with the dataset authors.

**Results:**

Our quantitative study suggests scattered effects of including categorical data for machine learning model training across predictive classes. Our qualitative analysis gives insights into how the categorical data were collected and why they were published, explaining some of the quantitative effects that we observed. Our findings highlight the social constructedness of categorical data in publicly available datasets, meaning that the data in a category heavily depend on both how these categories are defined by the dataset creators and the sociomedico context in which the data are collected. This reveals relevant limitations of using publicly available datasets in contexts different from those of the collection of their data.

**Conclusions:**

We caution against using data features of publicly available datasets without reflection on the social construction and context dependency of their categorical data features, particularly in data-sparse areas. We conclude that social scientific, context-dependent analysis of available data features using both quantitative and qualitative methods is helpful in judging the utility of categorical data for the population for which a model is intended.

## Introduction

### Background

Artificial intelligence (AI) promises to transform medical diagnostics and treatment; however, this paradigm change comes with social and ethical challenges [1,2]. One particular challenge is the persistent unreliability of medical machine learning models for diverse populations [3-7]. The variation in performance reliability across populations stems, among other factors, from data scarcity in medical datasets. Publicly available datasets to train medical models, compared to those in other disciplines, are small or have little variation, often featuring populations from a specific area of the world in which the data were collected. When a machine learning model is trained on such a dataset, the results can have limited generalizability to a more diverse population seeking medical care, having disadvantaging or discriminatory effects [8].

Computer scientists increasingly turn to innovative multimodal computational methods to counter data scarcity and leverage the full potential of the limited available information. Recently, (deep) multimodal data fusion algorithms (ie, algorithms incorporating available categorical data such as a patient’s medical history and demographics in addition to a main data category such as a medical image) have been developed and successfully used in, for example, dermatology and radiology [9-14]. However, the effects of including categorical data for machine learning training are understudied, particularly regarding models such as medical image classifiers intended to equitably serve individuals from diverse populations. However, understanding the impacts of categorical data on machine learning outcomes is crucial for the development of fairer and more accurate machine learning models.

From a social science perspective, using categorical data for machine learning training is controversial. Categorization, by definition, clusters individuals in groups based on their attributes. Many categories, such as gender, socioeconomic status, and skin color, are used broadly to structure everyday communication and facilitate scientific research. However, these categories are socially constructed, meaning that they are the result of artificial definitions that go beyond biological facts [15]. As a result, socially constructed categories are unstable and can be too broad or narrow for the context they are used in or simply inadequate shorthands [3]. When rendered statistically, such as in a machine learning model, the inexactitude of such categories becomes materially significant and can introduce biases.

This paper proposes a mixed methods approach to assess the qualitative utility of available categorical data before machine learning model training. We first assess data categories’ social construction as a major reason behind their context dependency. We then report an exploratory, quantitative examination of the effects of data features from a specific dermatological dataset’s [16] unique categorical data features on a vision transformer model. We square the quantitative findings with a qualitative study of the data collection process and inclusion and exclusion logics of the dataset’s categorical data for publication.

Our results suggest how data collection practices and publication decisions are roots for the observable effects of categorical data on machine learning model training. This study practically exemplifies how insights into the social construction and context dependency of available categorical data can be derived and used for inclusion or exclusion decision-making during training. This demonstrates how integrating quantitative and qualitative insights to decide on the inclusion or exclusion of categorical data can help incorporate health equity considerations into machine learning–based medical imaging workflows, such as considering who is served or neglected when certain data categories are used or disregarded during training. Such interdisciplinary results, we argue, are pertinent to inform model training accounting for the unavoidable performance differences of machine learning models for different groups.

### The Dermatological and Surgical Assistance Program at the Federal University of Espírito Santo 20 Dataset

In dermatology, the primary data category typically consists of images of skin lesions. Additional data often collected include the patient's age, sex or gender, and dermatologically relevant factors such as skin type or the location of the lesion on the body. We base our work on experiments with and analyses of the Brazilian Dermatological and Surgical Assistance Program at the Federal University of Espírito Santo (PAD-UFES) 20 dermatological dataset [16]. The dataset contains 2298 entries in total labeled for 6 skin lesion classes: 3 malignant (basal cell carcinoma [BCC], nevus [NEV], and melanoma [MEL]) and 3 benign (seborrheic keratosis [SEK], actinic keratosis [ACK], and squamous cell carcinoma [SCC]).

The PAD-UFES 20 dataset has a unique combination and a comparatively high number of 21 patient-focused data features. These features are leverageable for machine learning model training. The dataset’s data cover the patients’ medical information and history (eg, *skin_cancer_history*), medical details about the skin lesion not captured by the image (eg, *itch*, *grew*, *bleed*, and *elevation*), demographics (eg, *age*, *gender*, *background_father*, and *background_mother*), environmental determinants (eg, *pesticide*, *has_piped_water*, and *has_sewage_system*), and lifestyle (eg, *smoke* or *drink*), with many of the data types being categorical (ie, the assignment of individual patients to a category is a matter of definition; Table 1 [15]). This unique combination of data features for each entry, its comparably small sample size for a machine learning context, and its public availability that makes PAD-UFES 20 accessible for researchers worldwide were significant criteria for choosing this dataset for exploring the implications of using categorical data for machine learning training.

Examining the PAD-UFES 20 dataset’s categorical data features illuminates its theoretical limitations. Most of the samples in the dataset originate from Brazilian patients of central European ancestry. Prevalent characteristics of this group include a White phenotype and a dominantly disadvantaged socioeconomic status. The combined characteristics of lighter skin tones typical of the White phenotype and their heightened sun exposure during various activities common among those of a disadvantaged socioeconomic status in Brazil, such as fieldwork and domestic labor, make this subgroup particularly susceptible to skin lesions induced by the intense Brazilian sun. While the resulting high prevalence of skin cancer in the dataset renders the PAD-UFES 20 dataset effective for training a skin lesion classifier, especially for skin cancer, it is crucial to recognize that the dataset’s population is notably specific and, thus, introduces significant biases. Because the dataset is skewed toward lighter skin tones and a particular socioeconomic status, a model trained using this dataset might, even when trained on image data alone, limitedly generalize to other populations. In the following section, we will consider how data categories are socially constructed and, thus, context dependent, calling their utility for the model training process into question.

**Table 1:** Description of (categorical) data attributes available with the PAD-UFES 20 dataset (in the metadata CSV file).

Attribute	Description
patient_id	A string representing the patient ID – example: PAT_1234
lesion_id	A string representing the lesion ID – example: 123
image_id	A string representing the image ID, which is a composition of the patient ID, lesion ID, and a random number – example: PAT_1234_123_000
smoke	A Boolean to map if the patient smokes cigarettes
drink	A Boolean to map if the patient consumes alcoholic beverages
background_father and background_mother	A string representing the country in which the patient’s father and mother descends. Note: many patients descend from Pomerania, a region between Poland and Germany. Although it is not a country, we decided to keep the nomenclature, since they identify themselves as Pomeranian descendants.
age	An integer representing the patient’s age
pesticide	A Boolean to map if the patient uses pesticides
gender	A string representing the patient’s gender
skin_cancer_history	A Boolean to map if the patient or someone in their family has had skin cancer in the past
cancer_history	A Boolean to map if the patient or someone in their family has had any type of cancer in the past
has_piped_water	A Boolean to map if the patient or someone in their family has had any type of cancer in the past
has_sewage_system	A Boolean to map if the patient has access to a sewage system in their home
fitspatrick	An integer representing the Fitspatrick skin type
region	A string representing one of the 15 macro-regions previously described
diameter_1 and diameter_2	A float representing the skin lesions’ horizontal and vertical diameters
diagnostic	A string representing the skin lesion diagnostic – BCC, SCC, ACK, SEK, MEL, or NEV
itch	A Boolean to map if the skin lesion itches
grew	A Boolean to map if the skin lesion has recently grown
hurt	A Boolean to map if the skin lesion hurts
changed	A Boolean to map if the skin lesion has recently changed
bleed	A Boolean to map if the skin lesion has bled
elevation	A Boolean to map if the skin lesion has an elevation
biopsed	A Boolean to map if the diagnostic comes from clinical consensus or biopsy

### The Social Construction of Categorical Data

Data categories featured in publicly available datasets such as the PAD-UFES 20 and used to train medical imaging machine learning models are often assigned to someone or something as biological despite being “socially constructed.” Socially constructed categories are historically produced, reproduced, and changed within institutions or cultures [15]. Many demographics, for example, age, race, or gender, are examples of socially constructed categories. They play a key role in shaping and structuring human communication as well as the beliefs we hold about the world, but they are also loaded with meanings that go beyond their biological bases or initial definitions and depend on the working definitions of these categories in a specific assessment setting [15]. Hence, while sometimes appearing universally applicable and comparable, socially constructed categories harbor underlying ambiguities that make them context dependent. When such context-dependent data are used for machine learning training, their ambiguities create biases and can disadvantage population groups, often those already marginalized [3,5].

In the early nineties, Suchman [17], building on the analysis by Winner [18] of how artifacts have politics, described how categories have politics. The works by both Winner [18] and Suchman [17] drew attention to how moral values are inscribed in human-made constructs, be they artifacts made of concrete, such as bridges that disallow a specific population from entering a public beach [18], or categorial classifications, such as racial categories that group specific individuals and bestow upon these groups value-laden associations [17] that have tangible, measurable impacts on aspects of their lives, such as income, access to health care, and education [19-21]. The category “race” and its experienced disparities constitute an illustrative example of how categories are socially constructed and create social and political struggles for those classified under them. To elucidate, we can juxtapose the official “race” categories used in the census of countries such as the United States, Brazil, and Germany. In the United States, the collection of racial categories has undergone much change since the establishment of racial categories in the country’s census in 1790 [22]. Today, the US Office of Management and Budget “requires five minimum categories (White, Black or African American, American Indian or Alaska Native, Asian, and Native Hawaiian or Other Pacific Islander) for race [and] permits the Census Bureau to also use a sixth category - Some Other Race” [23]. Following Statistical Policy Directive 15 in October 1997, “the option to choose multiple racial categories was introduced as part of the U.S. government’s routine data-collection mission” [24]. For census data collection in Brazil, on the other hand, “white, brown, black, yellow (amarelo, i.e., of some Asian descent), and...the Indigenous (indígena) category [which was added] in the 1991 census” [25] constitute the “official” racial categories. In Germany, as another contrasting example, the census systematically omitted data collection of ethnic and racial categories after World War II. Instead, the German census collects “related data on foreigners, migrants and their descendants” [26], such as individuals’ “migration background.”

Note how the different handling of racial categories in a census creates subtle classification differences. Foremost, the categories collected are different. As a result, a single person could be classified differently depending on whether they live in the United States, Brazil, or Germany. Second, the categories themselves are based on different features. For example, “[u]nlike the USA, color or race in Brazil refers primarily to appearance rather than descent” [25]. Third, the approach to collecting the data is notably different. While, in the United States, the categorization was conducted by an authority employee until 1970 [22], “[s]elf-identification has been the official method for recording racial category membership in Brazil since 1950” [25]. Finally, Germany applies a “color blind” approach, collecting neither appearance-based nor self-identified racial data in its official census [26]. The decisions (not) to collect and how to collect the data shape the potential applications of the data on a country-by-country basis and influence the local medical data collection practices. The resulting differences hamper cross-national comparison and complexify the transfer of publicly available datasets that include such categories to different national contexts (it is important to note that the categories used for assessments in the official census do not always translate to the medical sector; for example, in the United States, the census categories are being challenged for the context of medical use by the Agency for Healthcare Research and Quality [27]). In addition to national differences in handling racial data categories, the inexactitude of racial categories and their intertwining with proxies such as skin color have been criticized [28]. In increasingly diverse populations, classifying someone as belonging to a single or even multiple races becomes increasingly complex and, thus, inaccurate. Because assessment strategies and categories can never be defined in a fine granular enough way, the categories are, by default, more descriptive of some than others classified within the same category. Following a recent controversy on a published paper that distinctly depicted human ancestry [29], a coauthor of the paper said that their “analysis reaffirms that race and ethnicity are social constructs that do not have a basis in genetics” [30].

In the context of machine learning models, the nuanced effects of using social constructs as categories can impact performance. Computer scientists who build machine learning and other statistical models “work every day on the design delegation, and choice of classification systems and standards” [24]. Thus, they have immense decision-making power over the specifications of the classification systems implemented. However, as Bowker and Star [24] note, “few see them as artifacts embodying moral and aesthetic choices that in turn craft people’s identities, aspirations, and dignity.” When, as a result, machine learning models process multiple individuals as classified under a single data category, subtle distinctions crucial for the model’s intended use might be overlooked, manifesting inaccuracies and biases. The resulting models will tend to perform better for those who align closely with the mean of their assigned category and less reliably for individuals who fall between or outside of the categories or are placed within a category that is too broad. Famous examples of the effects that the incautious consideration of racial categories can have in practice are the “racist soap dispenser” [31], which would not identify darker-skinned hands and, thus, not dispense soap for them; Google Photo’s facial recognition software, which identified darker-skinned individuals in photo postings as gorillas [32]; the perpetuation of discriminatory biases through search engines [33]; and the worrisome automation of decisions on whether someone is worthy of help, for example, when triggering child abuse investigations [34].

In health care, studies accounting for racial differences have reified categorial differences of inexact, socially constructed categories. For example, the hypertension medication BiDil was granted Food and Drug Administration approval after showing efficacy for Americans of African ancestry, whereas earlier, when tested on Americans of European ancestry, it was believed to be ineffective. Duster, a social scientist and critic of using socially constructed categories in genomic research, criticized the parties involved in both the study and its approval for insufficiently accounting for the “complex feedback loop and interaction effect between phenotype and social practices related to that phenotype” [35]. His work contributed to a large body of science studies scrutinizing the utility of assessing and using race, ancestry, gender, and other data categories in different areas of medical research [36-42]. For example, in precision medicine, scholars claimed that the absence of biomedical significance of categorizations creates shorthands, which are, at most, unhelpful for meaningful statistical inference [43,44]. Despite potentially providing clues for correlations, data categories used in health care are frequently proxies for other factors that matter in the context of diagnosis or treatment. In dermatology, health and its categorial social determinants are particularly intertwined as different skin types have different susceptibilities to skin lesions [45].

### Feature Selection: The Current State of the Art to Assess Data Feature Utility for Machine Learning

The fact that data features have different utility for different contexts is well established in the data science community. Hence, so-called feature selection methods have followed the introduction of techniques that use multiple, possibly multimodal (ie, various data types) data features in a single medical machine learning model [46]. Using feature selection in developing a machine learning model, computer scientists test which data features make sense to include for training. To do so, feature selection methods render available data features statistically and rank them regarding their relevance for the intended use of a particular model. In addition to helping choose which data to include for a model, data feature selection methods have been used to create new insights about certain statistical connections of variables and, thus, strengthen the explainability of a model [47] or find clues about the root causes of certain diseases [48].

The feature selection approach effectively provides insights into the statistical relevance of the data features of a particular dataset and its corresponding machine learning model. However, available feature selection methods fail to provide qualitative insights into why a specific data category contributes to a model’s reliability, explainability, and performance. An additional limitation of currently used feature selection methods is that they usually look at the relevance of a data category across the entire model and fail to investigate performance increases or decreases due to the inclusion or exclusion of certain data features per class. As a result, the effects of irrelevant data features, or those reducing the performance of a particular class, might be balanced out if another class does correlate with the same data features.

## Methods

The methods for this work were a mix of applied computing and qualitative investigations based on grounded theory [49]. For the claims we make, it is necessary to incorporate both computer scientific findings from experimenting with the dataset and qualitative accounts that can shed light on some of the effects we observed in more detail than numbers can.

### Quantitative Analysis

The quantitative analysis aimed to investigate the effect that each available data feature, including categorical data, has on a skin lesion classifier model to provide insights into the utility of each data feature. Therefore, we trained multiple similar transformer-based models using the PAD-UFES 20 dataset while randomizing one of the data categories in each case. We then compared the performance of the obtained models per class. Coauthors AW and TC-B led the method and implementation of the quantitative analysis.

For preprocessing, duplicates and entries with 2 images or lacking data were excluded. A total of 64.14% (1474/2298) of the entries remained. Patient data were first converted to categorical data (numerical data entries [age and lesion size] were binned) and encoded into soft one-hot vectors.

We trained identical multi-classifier vision transformer models (with a training, validation, and testing data split of 70%-15%-15%) based on the architecture published in the study by Cheslerean-Boghiu et al [13]. As the feature extractor, we used the “vit_small_patch16_224” vision transformer, which comprises 12 transformer encoder layers and a classification head operating on embedding vectors with 384 elements. The model, loaded via the PyTorch Image Models library [50] with ImageNet-1K (Standford Vision Lab) pretrained weights, operates at an input resolution of 224 × 224 pixels. Positional embeddings were also derived from ImageNet pretraining, and we performed fine-tuning across the entire model without any layer freezing. The framework used for this implementation was PyTorch.

One model was trained using all available data, and one model was trained using randomized values for all data features. For the other 16 models, we randomized the input for 1 data feature in each case: *Fitzpatrick*, *background_father*, *background_mother*, *gender*, *hurt*, *region*, *age*, *smoke*, *drink*, *pesticide*, *has_piped_water*, *has_sewage_system*, *cancer_history*, and *skin_cancer_history*. The data features we tested were selected based on an internal discussion about the potential utility of the data features for the model. These decisions were made before having spoken to the authors of the PAD-UFES 20 dataset.

To solidify our results, all models were trained in 5 separate runs each; the randomized values of the models were set differently in each training run. The seed was set differently in every run. The fixed input dimensions of our vision transformer model constituted the rationale for this process. Feature randomization has been successfully applied in other studies [51]. Leaving out data features instead of randomizing them would alter the model and, thus, impair our analysis of the effects of each data feature.

For the result presentation, we concatenated all runs per model (one model with all data features included, one model with randomized values for all data features, and one model for each randomized data feature). We created receiver operating characteristic (ROC) curve plots for each of the 6 classes (NEV, BCC, MEL, ACK, SCC, and SEK). Each ROC plot shows the performance of all models for one of the classes in color-coded graphs. For each performance representation, we included area under the ROC curve values and CIs calculated using the nonparametric bootstrap method with 10,000-fold resampling at the image level.

The quantitative part of our analysis is an exploratory study; needs replication to substantiate it, including reproduction with combinations of data features included or excluded; and is naturally limited by the small sample size of the dataset, particularly in combination with the transformer model architecture used. This setup was selected not to develop a robust skin lesion classifier but to simulate a real-world application of this dataset [13], highlighting the realistic limitations and implications associated with its use.

### Qualitative Analysis

The qualitative analysis featured in this work is based on one-on-one interviews with 3 of the authors of the PAD-UFES 20 dataset. The interviews aimed to gain (unpublished) insights into the dataset and the data therein.

The first author of this paper (TW) approached André Pacheco, assistant professor at the Federal University of Espírito Santo, lead of the PAD-UFES project, corresponding author of the dataset, and presenter of multiple (publicly available) talks on the PAD-UFES 20 dataset, via email, establishing first contact, explaining the context of the study, discussing participation in an interview for the study at hand, leading to the first interview in October 2023. After the initial interview, 2 other authors of the dataset publication were recruited via snowballing: Gabriel Giorisatto, a machine learning student working in André Pacheco’s research laboratory when the PAD-UFES project started and who helped gain skin cancer domain knowledge facilitating dialogues with medical colleagues, and Breno Krohling, a machine engineer who was working as an undergraduate student in the laboratory facilitating the PAD-UFES project by helping with data collection. The second and third interviews were conducted in October 2024.

An initial interview was conducted with André Pacheco in October 2023. To solidify the findings, 2 additional interviews were conducted in October 2024 (Breno Krohling and Gabriel Giorisatto). All interviews were conducted by TW via Zoom (Zoom Video Communications) and followed the peer-to-peer interview method [52] using a semistructured interview guide to structure the conversation. The interviews lasted between 40 minutes and 1.5 hours and covered the genesis of the PAD-UFES 20 project; the context in which the dataset was created; the authors’ rationale for collecting the data; their rationales for collecting each of the data features; the initial intended use of the dataset; how access to the community centrally featured in the dataset was established; how the dataset was created; and how the authors of the dataset reflected on the public use of the dataset, which, at least in some instances, includes the data features for analysis.

Initial interview transcripts were created with AI assistance [53]. TW double-checked and corrected the transcripts while relistening to the interview audio files. The interview transcripts were analyzed using a grounded theory–inspired coding process that included line-by-line coding and axial coding with sensitizing concept clusters according to initial findings [49]. Initial codes were selectively recoded to inform our analysis and condition our findings. TW created memos based on the codes that later formed the basis for the qualitative accounts in this work. The quotes used in this manuscript have been edited for readability and were returned to the interviewees to double-check that the correctness of meaning stayed intact before publication. The interview data showed strong consistency across participants, suggesting a high level of theoretical saturation.

We followed the COREQ (Consolidated Criteria for Reporting Qualitative Research) checklist for reporting the qualitative part of this study (Multimedia Appendix 1).

### Ethical Considerations

Before each interview, TW explained the reservations about participating in the study, including the interviewees’ ability to opt out of the study at any time and the impossibility of granting them pseudonymization. All interviewees gave their written informed consent to participate in the interviews and to be referred to by their real names throughout this manuscript. Interviewees did not receive any compensation for participating in the interviews.

## Results

### Overview

In the following sections, we present our findings of the experimental inclusion or exclusion of the data features of the Brazilian PAD-UFES 20 dermatological dataset for training a vision transformer multi-classifier and the pairing of these quantitative findings with a qualitative investigation. We observed scattered effects in the quantitative analysis and interpreted them regarding the creation and use practices of publicly available medical datasets. However, combined with our qualitative analysis, the observed scattered effects provide insights into how data collected in or for a particular social context can have adverse effects when used to train models intended for different or broader contexts. On the other hand, our data solidify evidence that the optimization of a model for a social context such as the dataset’s by including categorical metadata for training can make the model more precise if data categories are not too broad, narrow, or ambiguous or shorthands. However, there is reason to believe that, in these cases, the precision of the model overfits the dataset’s context and, hence, the resulting model might impede the ability of health care professionals to decide whether a particular model will work for a new patient. Our insights advise for interdisciplinary dialogue to assess the data or context fit before model training.

### Changes in Model Performance Through Data Feature Randomization

**Figure 1:**
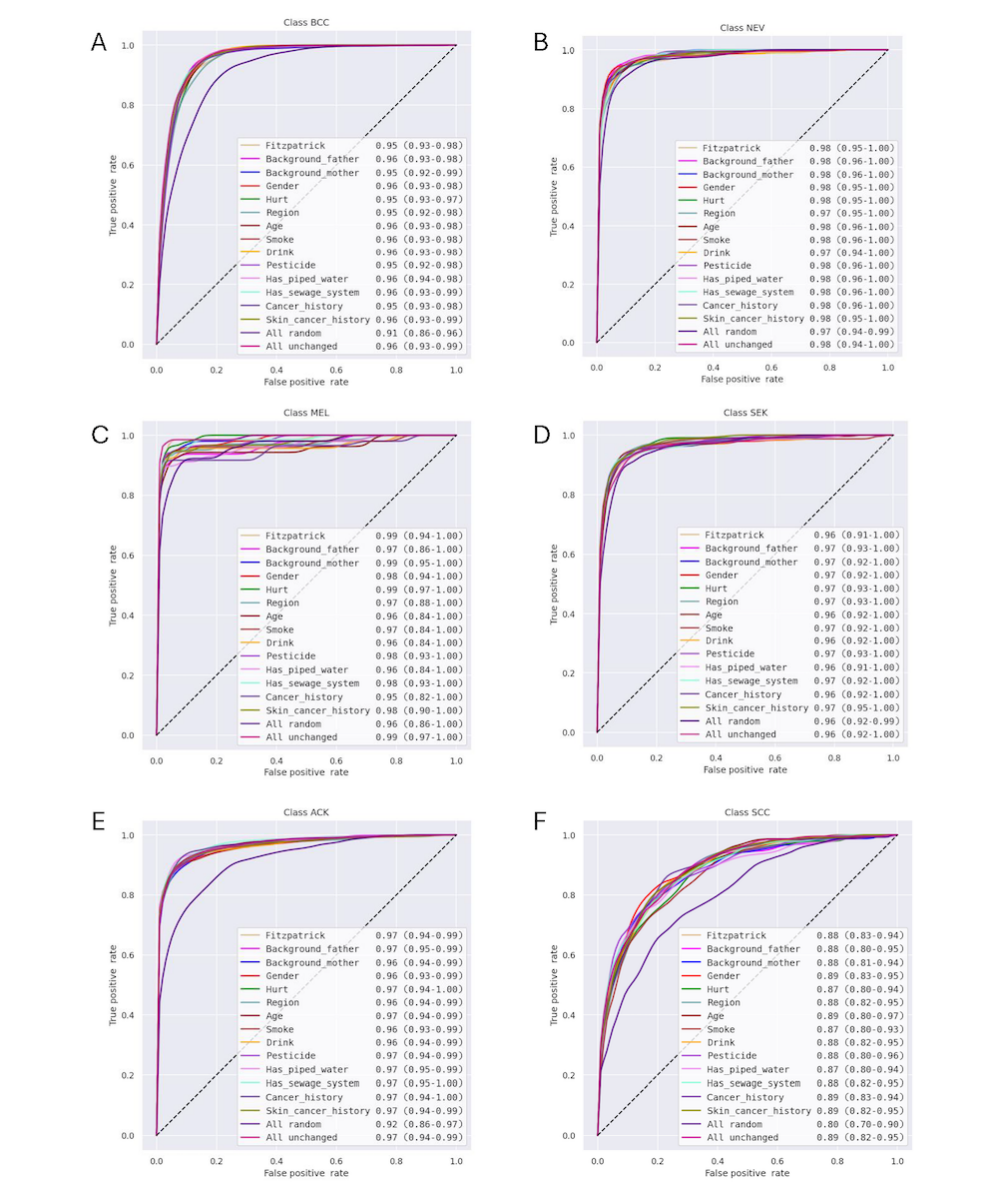
Performance of the vision transformer models trained on the Dermatological and Surgical Assistance Program at the Federal University of Espírito Santo 20 dataset per class—(A) basal cell carcinoma (BCC), (B) nevus (NEV), (C) melanoma (MEL), (D) seborrheic keratosis (SEK), (E) actinic keratosis (ACK), and (F) squamous cell carcinoma (SCC). Each graph represents the mean performance of 5 similar concatenated models; the color of each graph corresponds to the metadata feature that was randomized for this model’s training (see the legend in each of the images) except for the “all random” and “all unchanged” graphs. The “all random” graph represents the model performance when all metadata features were randomized, and the “all unchanged” graph represents the model performance when all metadata features were included in the training as per the dataset.

The juxtaposition of the performance of the models, which were trained using all available data features (model 1), using randomized values for all available data features in training (model 2), or randomizing 1 data feature in each case (models 3-16), showed scattered effects for each class (Figure 1).

#### Randomizing All Data Features

Across all classes, the performance of the models decreased when all data features were randomized for training compared to the model for which we included all data feature values according to the PAD-UFES 20 dataset (“all unchanged”), indicating the predictive utility of the data features for the classes.

The magnitude of the performance drop from the “all unchanged” to the “all random” model varied notably across classes. For example, for SCC (Figure 1F), the performance decreased from 0.89 to 0.80 by 0.09. For other classes, the performance difference was only very slight (eg, 0.01; Figure 1B), or the performance even stayed the same (eg, 0.00 for SEK; Figure 1D) when all data features were randomized. This indicates that the data are more relevant to some classes than others.

#### Randomizing Individual Data Features

Randomizing individual data features impacted the models. In some instances, the model’s performance decreased when a data feature was randomized, indicating that the randomized data feature had predictive utility for the model’s performance. For example, randomizing the “cancer history” data feature reduced the average predictive performance for the melanoma class (Figure 1C) by 0.04. Considering the breadth of some confidence intervals (CIs), this effect seems even more pronounced for some of the models created in our experiment. In other cases, the model’s performance increased when a data feature was randomized, indicating that the randomized data feature hampered the model’s predictive power. For example, when “background father” was randomized, the performance for the SEK class increased by 0.01 compared to that of the “all unchanged” model.

The effects observed in our quantitative analysis coincide with the qualitatively contextualized expectations regarding the relevance of data features to the overall model and some classes (detailed analysis in the following sections). As a general trend, the randomization of data features considered highly medically relevant by the dataset’s authors, on average, also decreased the model’s performance, indicating that the data features have predictive power. The performance variance varied per class and randomized data feature across those expected to be highly medically relevant (eg, *itch*, *bleed*, and *elevation*) and those expected to be confounded through their social context dependency to the envisaged prediction (eg, *background_mother* and *background_father*, *has_sewage_system*, or *has_piped_water*).

#### Comparative Analysis of the Randomization of Individual and All Data Features

When all data features were randomized, the model in some cases performed better than a model for which we randomized only 1 data category during training. This suggests that a model trained using all random data features ignored the meta data and took only the image data into account. The fact that the CI reaches (close to) 1.00 in most models supports this hypothesis and further shows that, in our experiment, relatively well-performing models were obtainable even when one or all data features were randomized. Note that, in our experiment, we validated the performance of the models on a split from the training dataset. The performance increased for some models when data were included, which can partially be explained by the heterogeneity of the PAD-UFES 20 dataset, which is largely biased toward a lighter-skinned immigrant population. The model likely overfits this population.

Overall, the quantitative analysis of the models showed a scattered impact of the available data features in the PAD-UFES 20 dataset, making it difficult to quantitatively evaluate the usefulness of including individual data features. However, medical data appeared slightly more pertinent to the model than other categories serving as proxies, whereas demographic, context-dependent variables showed uneven relevance per model class. However, note that the empirical evidence exploratively derived from our quantitative analysis can only provide limited (stand-alone) information about specific impacts of the inclusion and exclusion of data features from the PAD-UFES 20 dataset and is rather meant to showcase the potential of quantitative analysis for the evaluation of data features. Checking whether the results are consistent across other feature selection methods would exceed the scope of this exploratory study. Still, it would be highly relevant to future work to provide further insights into the utility of the PAD-UFES 20 data.

### Qualitative Contextualization

#### Overview

This section features qualitative data from interviews with the authors of the PAD-UFES 20 dataset to show an example contextualization of the quantitative findings using qualitative analysis. The accounts presented provide insights into how the dataset was created and why certain data categories were included in and excluded from the publicly available version. Thus, they provide insights into the quality of the publicly available PAD-UFES 20 data features.

#### Inclusion: How Data Categories Were Chosen

First, it is essential to understand how the team decided which data categories to include for data collection. Our interlocutors told us that one purpose of the PAD-UFES project was to digitize the university’s dermatological data collection process that, at the time, was still conducted manually by medical students. Therefore, an initial set of data categories to collect originated from formerly institutionally established data collection practices. The refinement of the data collection specifications for the PAD-UFES 20 dataset specifically was established while aiming to satisfy multiple actors’ interests at the same time:

They [the medical colleagues] wanted to have this data to make research on this data, so, for example, the geographic location of the patient, if the patient has clean water, the income of the patient per month...they wanted relevant data for their research about the demography of the patients. This information was initially not relevant to us, we wanted specific information about the lesion. So, we had these two types of data to collect.

Gabriel

Hence, some data categories were defined independently by medical students and scholars, whereas the computer scientists leading the data collection were less interested in these.

To determine which data categories would be relevant for machine learning, André and his colleagues referred to the “reporting standard in most medical data sets” (Breno) and consulted additional medical experts. André explained that their selections of which data features to collect were ultimately related to the following:

...specific questions [dermatologists ask their patients], like “does this lesion itch” or “has it bled in the past”...“has this been growing”...this kind of questions. Because they [doctors] try to simulate the algorithm that they have in their head.

André

On the basis of the factors that dermatologists reported working with to diagnose a lesion, André and his colleagues created a combination of data categories to potentially accompany the dermatological images. They ran the categories by the dermatologists again before starting the data collection process, asking the following:

If you have only this information, can you classify or...provide a diagnosis from this lesion for this lesion? And most doctors said “okay, now I have enough information.”

André

#### Re-Exclusion: Medically Irrelevant Data

Despite basing the choice of which data categories to collect on dermatological clinical practice, it became clear during data collection that not all data categories seemed reasonable to include for analysis:

[For the dataset creation] we asked people if they had contact with chemicals. Most of them work on farms. They are peasants...and some doctors say chemicals, you know, when you put them in the food [on the fields], they may have an impact on skin lesions. So, we have this in the data set. But most doctors don’t ask [their] patients [about chemicals during skin assessments] because they don’t think this is relevant.

André

The “pesticide” category serves as a representative example here. Gabriel and Breno shared similar insights regarding the categories “has_sewage_system” and “has_piped_water.” They reported that these categories were initially collected for medical students to analyze demographic factors and skin cancer or explore potential causal links between these features and skin cancer that could not be established in a subsequent analysis [54]. Notably, they still chose to publish these data categories despite excluding them from their machine learning analysis after deeming them medically irrelevant:

We left this information there [in the dataset] because we thought something like “maybe someone in the world wants to have this information to do something with it,” right?

André

#### Re-Exclusion: Unreliable Answers

Some other data categories available in the dataset were unreliable due to ambiguous internal definitions or varying degrees of patient honesty, such as the following:

If the patient uses cigarettes or drinks...if they consume alcohol. Those are general questions most people don’t like to answer.... Most of them say no, but sometimes they use that.

André

André explained that the resulting vagueness of the data features “smoke” and “drink” caused them to disregard these categories in the version of the dataset that they used for training algorithms. However, again, they held onto these features for publication of the dataset in case “someone wants to do some analysis with it,” as Breno said. This highlights the importance of critically reflecting on the available data features for their applicability for the intended use of a new model and the integrity of the categories.

#### Re-Exclusion: “Purposeful” Shorthands

Particularly striking data categories in the PAD-UFES 20 dataset are “background_father” and “background_mother.” André and his colleagues included these data categories for data collection for the following reason:

...many people in Brazil are descendants from Europe. Immigration. So, you know, this is a complicated feature because if I ask [a patient about their background and they say] “Oh, I’m descended from Germany,” we know that they are very white. They probably have Fitzpatrick skin type 1 or 2...because they work on a farm in the sun.... [Sun] exposure and being white is a very, very, um, you know, predisposition for skin cancer.

André

Hence, the categories referring to the patients’ parents’ background are conscious shorthands to assess their skin color. When asked why they collected both the patients’ parents’ backgrounds and their Fitzpatrick skin type, André answered the following:

Because sometimes people don’t know from which part of the world they descend. And at the office, they use that for statistics...just to see which region [the patients come from]. And in Brazil, there is a very high miscegenation. It doesn’t mean if people descend from, I don’t know, Italy, for example, [some might be] very white and [others], they have, you know, Fitzpatrick skin type 3 or 4 because of miscegenation, right? So, it tells us some part of the history, but not the whole history.

André

Several noteworthy observations emerge from this and similar accounts that we obtained from our interlocutors. First, it is interesting to highlight the decision to assess the migration background instead of racial categories. The rationale for including the background categories seems closely related to race-related concepts. However, the team hoped to assess who migrated from countries in which lighter skin tones are more prevalent. Second, while race or family origin appears to be an intuitive category to ask about to assess skin tone, its medical relevance in the context of a dermatological classifier seems questionable under closer scrutiny. Individuals descending from a specific region may have a higher likelihood of a particular skin type, yet their actual skin characteristics could deviate significantly, especially if their parents themselves underwent migration. Finally, including the background categories served multiple purposes: assessing the skin type, potentially including genetically important information that goes beyond skin type, and delivering statistics. The assessment of the patients’ parents’ backgrounds is a shorthand to fulfill all these goals simultaneously; however, not asking these questions separately compromises the exactitude of the categories.

These nuances suggest that the “fitzpatrick_skin_type” variable might offer greater reliability in assessing the intended parameters of the background data categories. However, according to André, the team included the variable “fitzpatrick_skin_type” only as a backup to the background categories. They did so because they expected that some patients might be unable to provide satisfactory answers regarding their backgrounds or migration history. Furthermore, he argued, “Fitzpatrick is subjective. You know, maybe a doctor may say this person is type two, and another doctor says this person is type three.” Moreover, the Fitzpatrick skin type scale, being a social construct in itself, harbors numerous limitations regarding its suitability to assess the intended parameters. For example, the scale has recently been criticized for having a Eurocentric bias toward lighter skin tones [55] and for incorrectly and inadequately reflecting sunburn and tanning risks for skins of color [56]. A useful data category would assess a cause factor of the classes the dataset is used to predict. For example, assessing how much melanin a patient’s skin holds might be more expedient in the case at hand.

Therefore, it was not surprising that all interviewees told us that the project team ultimately did not use some of the categorical data features for their analyses. This shows, once again, how limited the utility of some of the data categories included in the publicly available dataset can be. The fact that, in their qualitative analysis, they “didn’t find [some features] that useful when compared to the other data features” (André) is in line with the scattered effects of our quantitative results, in which the utility of other data features was much more visible compared to that of the background categories (Figure 1). Again, this emphasizes the necessity for thoroughly scrutinizing the available data categories in publicly available datasets.

Another purposeful shorthand that André and his colleagues used can be found in the categories “has_piped_water” and “has_sewage_system.” The team told us that they collected these data for the following reason:

...most people [in the dataset] are very poor. They work in farms, you know, this kind of thing. Right? When you collect “has_piped_water” and “has_sewage_system,” you can draw some correlations to family income. Usually, people with low family income don’t have piped water and don’t have a sewage system. And they usually have more skin lesions.

André

Our other interlocutors seconded this, and Breno added that “the age was also important because, as...they were farmers, the older they were, the more sun-exposed they were, so they have an increased risk of skin cancer,” showing how sometimes combinations of categorical data features were believed to have statistical importance for assessing skin cancer using predictive machine learning models.

However, as André noted, “this is a correlation, not causation.... They [poorer people] usually have more skin lesions because they don’t have money to afford the private treatment.” While it seems meaningful to assess social determinants of health, such as socioeconomic status, our aforementioned quantitative analysis did not show a particularly pronounced utility of this data feature across classes. In addition, André told us that these categories are not used in their model. Furthermore, because of the specific population in the dataset, correlations between the data categories are expected. For example, during data collection, André and his colleagues recruited heavily among the European immigrant population, creating a dataset predominantly of individuals with lighter skin tones and who were socioeconomically disadvantaged and prone to skin lesions because of their high sun exposure. While the research group is still grappling with mitigating the resulting biases, André pointed out that “it’s very hard to see how it could generalize to other groups here in Brazil.” When taken into consideration in isolation, some biases of a skin lesion classifier trained using the PAD-UFES 20 dataset might be productive for the accuracy of diagnoses in a population with similar characteristics to those of the population included in the PAS-UFES 20. According to André, the population they use their current model with—which is similar to the population of the dataset—is “98%...skin types under four.”

These Fitzpatrick skin types (1 to 3) are very well represented in the dataset; hence, the model they use regarding this aspect presumably works well for the vast majority of the patients they diagnose with the help of the model. This shows how a machine learning model might be locally helpful but hardly transferable to another context, in this case, a more diverse population, providing yet another reason for a context-sensitive assessment of data categories before their inclusion in training. However, what this leaves open is how the remaining 2% of patients in the Brazilian case can be granted fair access to dermatological care, how high the number of individuals who do not have access to this dermatological workflow is, and what skin types they have.

#### Renomination: Missing Categories Create Missing Values

André and his colleagues not only omitted specific data features from their model during analysis, but in other instances, they also discovered during the data collection process that specific data categories are indeed pertinent yet they had overlooked assessing them:

For example, the “elevation” data...one year after the [data] collection started, some doctors said to us, “Oh, the elevation is important, because if the lesion has elevation...it’s probably not a melanoma.” And we said, “Oh my God, this is very important information. Why haven’t any doctors here said that to us before.” Right. So, if you check the data set, you will see that elevation is missing for many samples in the dataset.

André

When the data features that result from the late inclusion of categories in data collection are included for training a machine learning model, the missing values must be accounted for; otherwise, they could distort the results. We did not explicitly test for the impact of missing values; however, because they are relatively many in the case of the PAD-UFES 20 dataset’s “elevation” category, it seems best to exclude this data category for training or wait for a new version of the dataset, which, according to André at the time of this writing, will be published shortly.

#### Publication: Making the Data Available to the Public

Our qualitative analysis showed that the authors of the PAD-UFES 20 dataset were aware of the caveats of the dataset. André and his colleagues carefully considered which data to include for the training of their model based on the quantitative analysis they performed and, importantly, the contextual knowledge they had about the population; the ways in which the data were assessed; and the quality of the data features they produced regarding unreliable answers, shorthands, medically irrelevant data, or missing values. However, when prompted about whether they had concerns about how their publicly available dataset might be decontextualized when used in other areas of the world by other developers with less contextual knowledge or applied to different population groups, André answered the following:

No, we don’t have concerns about that. We just released the data set to, you know, to help the research community. You know, when we started to work in this field, we felt like, “oh, the data, there are just a few open [publicly available] datasets, and it would be great if we could release a data set that people from any part of the world could use.” Right. But, of course, that can be used for good or bad. Of course, we cannot control what people do with this data set.

André

This account brings together several arguments. Again, it highlights that the team’s motivation to publish the PAD-UFES 20 dataset was to counter the frustration within the medical machine learning community regarding the challenge of accessing datasets just above the quantitative threshold for being effective for machine learning applications. While emphasizing the pervasive scarcity of data in health care and the urgent need for comprehensive datasets, all interviewees underscored the dual use potential of datasets—the obtained results can either be advantageous or detrimental for individual patients or society at large depending on the decisions made by those using the dataset.

On the basis of our analysis, the potential damages that uncontextualized datasets might result in are not necessarily due to adverse decision-making of developers but can stem from a challenging-to-bridge gap in contextual knowledge. The significant gap between the pressing need for publicly available data to pursue the promised benefits of AI in health care and the potential for unintended harms highlights the need to exercise caution when dealing with unfamiliar, publicly available categorical data. It prompts important questions about responsible data publication practices.

## Discussion

### Principal Findings

For this study, we conducted a quantitative analysis and interpreted it regarding the creation and use practices of publicly available datasets. Our results showed that vague data categories, which are medically irrelevant, unreliable, shorthands for other categories, or otherwise distorted (eg, by missing values), can impair the quality of a machine learning model either overall or for specific predictive classes. The second part of our study—a qualitative analysis of an interview with authors of the dataset—provided evidence that the effects observed in the quantitative study are due to the social context dependency of the data categories chosen for a dataset and the medico-social context in which the data were collected.

Our results suggest that the uncritical use of context-dependent categorical data from publicly available datasets can introduce significant biases. These biases are particularly challenging to detect and mitigate because, under current machine learning standards, performance tests—even subgroup-specific performance tests—are typically conducted on a test split derived from the same dataset used for training. As this test split shares its context-dependent categorizations with the training split, these biases can go unnoticed. Such biases pose a particular risk in sensitive fields such as health care, which are challenged by scarce data landscapes combined with high-stakes decision-making in which every false negative and false positive can be detrimental to individual patients. As we demonstrated, a qualitative examination of how categorical data features are defined and an assessment of where categories may be vague, unreliable, oversimplified, or otherwise unsuitable for the model’s intended application can help detect these biases.

### Limitations

It is important to note that the magnitude of the observed effects of data categories on a machine learning model’s performance likely varies depending on the dataset used. We expect, for example, that, if a similar study to ours were conducted using a larger dataset, the impacts of the data categories and washout effects would make the results more challenging to quantify. Conversely, in our experiment, the washout was less pronounced, and thus, the impact of the data categories became visible. However, the fact that we worked with a publicly available medical imaging dataset positions our results to raise significant concerns, particularly for domains such as medicine in which data scarcity frequently results in particularly small datasets being used for machine learning training. However, other studies should replicate our findings and investigate the impact of data categories in different settings.

An established limitation of case-sensitive research is limited generalizability. We would like to highlight that the phenomena observed in this study might not immediately lend themselves to generalization. This work intended to explore the interplay among data collection practices, the social construction of categories, and current use practices of publicly available datasets for machine learning model training in high-risk areas such as health care. While offering insights that might apply to similar cases or broader trends, this work, instead of deriving generalizable claims, exemplifies our research approach. We recommend conducting additional analyses when applying the approach to other scenarios. We made corresponding remarks throughout the manuscript. In line with the trend toward structured assessment of data categories [9-14], our data suggest that including well-defined and contextually relevant data features in machine learning training can increase the accuracy of a model. However, this renders overfitting likely, and the translation to a more diverse population likely holds potential for harm because the accuracy of a model for any given patient and, therefore, the reliability of the model’s outputs become more complex to assess. Further investigations are required to validate these hypotheses.

### Relationship to Prior Work and Outlook

Our results deepen concerns raised by scholars about the social construction of attributes and intersectionality, for example, that machine learning puts specific, already marginalized groups at risk of medical under- or overtreatment [3,5-7]. Following the idea that categories have politics [17,18,24], our analysis of the politics of data categories extended established risks of the use of image data [7] and cautions against the inconsiderate use of categorical data accompanying image data in medical machine learning settings and beyond. In light of the context dependency and social constructedness of categorical attributes, such as racial categories used in the official censuses of individual countries [22,23,25-28], our results highlight the need for sociomedical studies to identify more precise data categories tailored to each of the diverse contexts in which medical machine learning models are applied. We believe that an increase in data category quality is essential to fulfill the potential of enhancing machine learning algorithms by incorporating relevant data categories during training. To achieve this, interdisciplinary research that establishes context-dependent data category definitions is needed. Context-dependent definitions should account for the, as Duster [35] put it, “complex feedback loop and the interaction effect between phenotype and social practices related to that phenotype.”

As we have pointed out in this paper, the trend toward structured assessment of data categories in medical imaging is accompanied by an increase in data fusion algorithms. Both practices follow a sublime “more data is better” narrative previously reported in the context of publicly available image databases such as ImageNet [57]. This logic incentivizes those collecting data to include categories that fulfill multiple purposes simultaneously and those who develop machine learning models to include more of these potentially vague or too specific data features for training. Feature selection methods [46], the insights of which are based on statistics leveraged from the training dataset and lack qualitative contextualization, seem to be toothless against the context-sensitive limitations of data inclusion and exclusion for training. Additional mechanisms are needed to investigate the suitability of data categories for a new model, its context, and the population to which it will be applied. Consequently, this work advocates for deepening transdisciplinary collaborations in high-risk areas such as machine learning for medical images. We believe that the combination of applied computing and qualitative assessment is pertinent to assessing the contextual dependencies of data assessments and data applications and providing analytical insights into relevant gaps. As demonstrated in this research, considering quantitative and qualitative evaluations together can yield valuable recommendations for using or refraining from using data categories.

### Conclusions

This work case-sensitively explored the effects of including categorical data features for training in data-scarce and community-serving areas such as medical imaging. We suggested a mixed methods approach, a blend of quantitative and qualitative analysis, to draw contextual conclusions about the social construction and context dependency of categorical data in publicly available datasets before using them for machine learning training. We exemplified our approach by applying it to the PAD-UFES 20 dermatological dataset, for which we quantitatively observed scattered effects on model performance when comparing scenarios in which we included all available data features for training, randomized all data features, or randomized a single data feature at a time. A qualitative analysis of the genesis of the dataset revealed root causes for the futility of some of the available data features.

Highlighting the nuanced and complex relationship between categorical data features and model performance, our observations suggest the need for an overall cautious use of publicly available categorical data for machine learning training. Our findings showed that data collection and categorization practices are not always intentional (enough) and that data categories’ social constructedness and context dependency limit their transferability to new models. This underscores the importance of conducting qualitative, context-dependent analyses when considering integrating categorical data features into model training, particularly in domains in which the impact on diverse populations is paramount.

We further deduce from these findings that, first, interdisciplinary social science and health care scholarship is needed to create more purposeful and context-dependent definitions of data categories to guide data collection. Second, dataset creators should include detailed descriptions of the collection context of each data category. Finally, those using publicly available datasets should assume that more features may be available than are necessarily useful to include in training. Machine learning scientists should further institute robust practices to test available categorical data for their integrity within the context of the intended model application to prevent the perpetuation of biases when too narrow or vague data categories translate to their model. Importantly, the absence of reliable methods to assess and mitigate the adverse effects of context-sensitive data features creates an ethical imperative of refraining from including categorical data features unless proven safe for use in a diverse population related to the model’s intended use.

We believe our findings to be particularly relevant for fields such as medicine, where sample sizes are often limited, demanding meticulous scrutiny of available data features for suitability before incorporation into model training. We advocate for an interdisciplinary approach that combines technical rigor with sociological understanding, ensuring that machine learning models are developed with the complexity of categorial attributes in mind. This collaborative effort is essential for advancing the ethical and effective deployment of AI in a manner that respects the complexities of human societies.

## Appendix

Multimedia Appendix 1 COREQ (Consolidated Criteria for Reporting Qualitative Research) checklist for reporting the qualitative part of this study.

## Abbreviations

AI: artificial intelligence
COREQ: Consolidated Criteria for Reporting Qualitative Research
PAD-UFES: Dermatological and Surgical Assistance Program at the Federal University of Espírito Santo
ROC: receiver operating characteristic
SCC: squamous cell carcinoma
SEK: seborrheic keratosis

## Data Availability

The datasets generated during and analyzed during this study are not publicly available to protect the confidentiality and privacy of the participants but are available from the corresponding author on reasonable request. The Dermatological and Surgical Assistance Program at the Federal University of Espírito Santo 20 dataset analyzed during this study is available in the GitHub repository [58]. The architecture used to perform the analysis is available as a preprint [13].

## References

[1] Čartolovni A, Tomičić A, Lazić Mosler E (2022). Ethical, legal, and social considerations of AI-based medical decision-support tools: a scoping review. Int J Med Inform.

[2] Chen IY, Pierson E, Rose S, Joshi S, Ferryman K, Ghassemi M (2021). Ethical machine learning in healthcare. Annu Rev Biomed Data Sci.

[3] Buolamwini J, Gebru T (2018). Gender shades: intersectional accuracy disparities in commercial gender classification. Proceedings of the 1st Conference on Fairness, Accountability and Transparency.

[4] O'Neil C (2016). Weapons of Math Destruction: How Big Data Increases Inequality and Threatens Democracy.

[5] Benjamin R (2019). Race After Technology: Abolitionist Tools for the New Jim Code.

[6] Broussard M (2023). More Than a Glitch: Confronting Race, Gender, and Ability Bias in Tech.

[7] Obermeyer Z, Powers B, Vogeli C, Mullainathan S (2019). Dissecting racial bias in an algorithm used to manage the health of populations. Science.

[8] Azar AK, Draghi B, Rotalinti Y, Myles P, Tucker A (2023). The impact of bias on drift detection in AI health software. Proceedings of the 21st International Conference on Artificial Intelligence in Medicine.

[9] Cui C, Yang H, Wang Y, Zhao S, Asad Z, Coburn LA, Wilson KT, Landman BA, Huo Y (2023). Deep multimodal fusion of image and non-image data in disease diagnosis and prognosis: a review. Prog Biomed Eng (Bristol).

[10] Kline A, Wang H, Li Y, Dennis S, Hutch M, Xu Z, Wang F, Cheng F, Luo Y (2022). Multimodal machine learning in precision health: a scoping review. NPJ Digit Med.

[11] Praveen Kumar S, Sridevi S (2021). Image fusion algorithm for medical images using DWT and SR. Proceedings of the International Conference on Artificial Intelligence and Smart Systems.

[12] Zhang Y, Sheng M, Liu X, Wang R, Lin W, Ren P, Wang X, Zhao E, Song W (2022). A heterogeneous multi-modal medical data fusion framework supporting hybrid data exploration. Health Inf Sci Syst.

[13] Cheslerean-Boghiu T, Fleischmann ME, Willem T, Lasser T (2024). Transformer-based interpretable multi-modal data fusion for skin lesion classification. arXiv. Preprint posted online on April 3, 2023.

[14] Kharazmi P, Kalia S, Lui H, Wang ZJ, Lee TK (2018). A feature fusion system for basal cell carcinoma detection through data-driven feature learning and patient profile. Skin Res Technol.

[15] Kang M, Lessard D, Heston L, Nordmarken S (2017). Social constructionism. Introduction to Women, Gender, Sexuality Studies.

[16] Pacheco AG, Lima GR, Salomão AS, Krohling B, Biral IP, de Angelo GG, Alves FC Jr, Esgario JG, Simora AC, Castro PB, Rodrigues FB, Frasson PH, Krohling RA, Knidel H, Santos MC, do Espírito Santo RB, Macedo TL, Canuto TR, de Barros LF (2020). PAD-UFES-20: a skin lesion dataset composed of patient data and clinical images collected from smartphones. Data Brief.

[17] Suchman L (2013). Do categories have politics? The language/action perspective reconsidered. Comput Supported Coop Work.

[18] Winner L (1980). Do artifacts have politics?. Daedalus.

[19] Bailey SR, Saperstein A, Penner A (2014). Race, color, and income inequality across the Americas. Demogr Res.

[20] Ohlson M (2020). Effects of socioeconomic status and race on access to healthcare in the United States. Perspectives.

[21] Budig MJ, Lim M, Hodges MJ (2021). Racial and gender pay disparities: the role of education. Soc Sci Res.

[22] Strmic-Pawl HV, Jackson BA, Garner S (2017). Race counts: racial and ethnic data on the U.S. census and the implications for tracking inequality. Sociol Race Ethnicity.

[23] Race. U.S. Census Bureau.

[24] Bowker GC, Star SL (2000). Sorting Things Out: Classification and Its Consequences.

[25] Bailey SR, Telles EE (2006). Multiracial versus collective Black categories: examining census classification debates in Brazil. Ethnicities.

[26] Will AK (2019). The German statistical category “migration background”: historical roots, revisions and shortcomings. Ethnicities.

[27] Race, ethnicity, and language data: standardization for health care quality improvement. Agency for Healthcare Research and Quality.

[28] Jablonski NG (2021). Skin color and race. Am J Phys Anthropol.

[29] All of Us Research Program Genomics Investigators (2024). Genomic data in the All of Us Research Program. Nature.

[30] Kozlov M (2024). ‘All of Us’ genetics chart stirs unease over controversial depiction of race. Nature.

[31] This 'Racist soap dispenser' at Facebook office does not work for Black people. YouTube.

[32] Grant N, Hill K (2023). Google’s photo app still can’t find gorillas. And neither can Apple’s. The New York Times.

[33] Noble SU (2018). Algorithms of Oppression: How Search Engines Reinforce Racism.

[34] Eubanks V (2018). Automating Inequality: How High-Tech Tools Profile, Police, and Punish the Poor.

[35] Duster T (2005). Medicine. Race and reification in science. Science.

[36] Duster T (2015). A post-genomic surprise. The molecular reinscription of race in science, law and medicine. Br J Sociol.

[37] Epstein S (2010). Inclusion The Politics of Difference in Medical Research: (Chicago Studies in Practices of Meaning).

[38] Caulfield T, Fullerton SM, Ali-Khan SE, Arbour L, Burchard EG, Cooper RS, Hardy BJ, Harry S, Hyde-Lay R, Kahn J, Kittles R, Koenig BA, Lee SS, Malinowski M, Ravitsky V, Sankar P, Scherer SW, Séguin B, Shickle D, Suarez-Kurtz G, Daar AS (2009). Race and ancestry in biomedical research: exploring the challenges. Genome Med.

[39] Reardon J (2017). The Postgenomic Condition: Ethics, Justice, & Knowledge after the Genome.

[40] Collins FS (2004). What we do and don't know about 'race', 'ethnicity', genetics and health at the dawn of the genome era. Nat Genet.

[41] Kowal E, Llamas B (2019). Race in a genome: long read sequencing, ethnicity-specific reference genomes and the shifting horizon of race. J Anthropol Sci.

[42] Osborne NG, Feit MD (1992). The use of race in medical research. JAMA.

[43] Kenney M, Mamo L (2020). The imaginary of precision public health. Med Humanit.

[44] Lee S, Wailoo K, Nelson A, Lee C (2012). Waiting on the promise of prescribing precision: race in the era of pharmacogenomics. Genetics and the Unsettled Past: The Collision of DNA, Race, and History.

[45] Adamson AS, Smith A (2018). Machine learning and health care disparities in dermatology. JAMA Dermatol.

[46] Remeseiro B, Bolon-Canedo V (2019). A review of feature selection methods in medical applications. Comput Biol Med.

[47] Singh A, Sengupta S, Lakshminarayanan V (2020). Explainable deep learning models in medical image analysis. J Imaging.

[48] Strobl E, Lasko T (2022). Identifying patient-specific root causes of disease. Proceedings of the 13th ACM International Conference on Bioinformatics, Computational Biology and Health Informatics.

[49] Charmaz K (2006). Constructing Grounded Theory.

[50] Ross Wightman. GitHub.

[51] Adebayo J, Gilmer J, Muelly M, Goodfellow I, Hardt M, Kim B (2024). Sanity checks for saliency maps. arXiv. Preprint posted online on October 8, 2018.

[52] Müller R, Kenney M (2014). Agential conversations: interviewing postdoctoral life scientists and the politics of mundane research practices. Sci Culture.

[53] Amberscript homepage. Amberscript.

[54] Pacheco AG, Krohling RA (2020). The impact of patient clinical information on automated skin cancer detection. Comput Biol Med.

[55] Lasisi T (2021). The constraints of racialization: how classification and valuation hinder scientific research on human variation. Am J Phys Anthropol.

[56] Goon P, Banfield C, Bello O, Levell NJ (2021). Skin cancers in skin types IV-VI: does the Fitzpatrick scale give a false sense of security?. Skin Health Dis.

[57] Denton E, Hanna A, Amironesei R, Smart A, Nicole H (2021). On the genealogy of machine learning datasets: a critical history of ImageNet. Big Data Soc.

[58] labcin-ufes / PAD-UFES-20. GitHub.

